# Recurrent myxoinflammatory fibroblastic sarcoma: a case report

**DOI:** 10.1002/ccr3.949

**Published:** 2017-04-20

**Authors:** Niveditha Jagadesh, Daniel H. Miller, William Schenk, Steven Attia, Courtney E. Sherman, Cherise Cortese, Byron C. May, Robert C. Miller

**Affiliations:** ^1^ Department of Radiation OncologyMayo ClinicJacksonvilleFlorida; ^2^ Department of RadiologyMayo ClinicJacksonvilleFlorida; ^3^ Department of Hematology/OncologyMayo ClinicJacksonvilleFlorida; ^4^ Department of Orthopedic SurgeryMayo ClinicJacksonvilleFlorida; ^5^ Department of PathologyMayo ClinicJacksonvilleFlorida

**Keywords:** Myxoinflammatory fibroblastic sarcoma, radiotherapy, sarcoma, surgical resection

## Abstract

Myxoinflammatory fibroblastic sarcoma is a rare sarcoma which typically presents in the extremities and is treated by definitive surgery. In recurrent disease, the reported utilization of radiotherapy is increasing, and more modern techniques such as intensity‐modulated radiotherapy may be improving long‐term outcomes.

## Introduction

Myxoinflammatory fibroblastic sarcoma (MIFS) is a new and abnormal growth of mesenchymal tissue, typically involving the hands or feet, and can be either benign or malignant 1, 2. This classification of sarcoma belongs to a heterogenous category characterized by the presence of mucoid/myxoid extracellular matrices 2. Within this category, similar histological findings and the frequent lack of distinct immunohistochemical features make pathologic classification challenging. This often translates into difficulty when deciding on the best course of clinical treatment 3, 4. The first reports of MIFS in the medical literature appeared in 1998, and the tumor was described as a mixture of ganglion‐like cells interspersed with spindled and epithelioid cells on the background of inflamed myxoid and fibrosclerotic stroma 1. Such tumors are typically found in subcutaneous tissues as either single or multiple nodules near fibrous connective tissue regions of fat, fascia, or tendon sheaths 1. The tumor grows slowly and painlessly, generally carrying little potential to become metastatic 1, 4. Histological sections of myxoid matrices of the tumor contain inflammatory cells, including lymphocytes, plasma cells, neutrophils, and eosinophils 4. Grossly, its appearance is that of a poorly circumscribed gray mass, ranging from myxoid to hard in consistency, with characteristic focally necrotic and/or hemorrhagic areas 5.

On MRI imaging, most lesions demonstrate low signal intensity on T1‐weighted images and high intensity on T2‐weighted images. Postcontrast enhancement shows a wider spectrum of variability from relatively diffuse enhancement to more subtle linear and nodular peripheral enhancement, which often corresponds to areas of osseous involvement 1. The limited diagnostic imaging details available for MIFS tumors may make it difficult to distinguish this entity from other pathologic entities including giant cell tumor, or even begin cystic lesions. Lang et al. drew attention to the notion that heterogenous hyperintense areas may be found in conjunction with focal hypointense areas on T2‐weighted sequences, yielding the potential for confusion with the characteristics of giant cell tumors 6. The difficulty that exists in identifying MIFS tumors was reflected in a case of a 53‐year‐old female who was diagnosed with a smooth, painless, cystic mass of 2 × 2 cm on her right ankle 7. The lesion was described as being hypointense with a homogenous internal structure and regular margin characteristic of a benign and cystic lesion 7. An irregular hypointense area present in the center of the lesion motivated total excision, which confirmed the diagnosis 7.

An institutional and database review, with median follow‐up >2 years, demonstrated a rate of local recurrence of 22%, with a median time to recurrence of 15 months 5. Although MIFS is generally regarded as a low‐grade malignancy, recurrent disease has been shown to behave more aggressively with a locally destructive nature and the ability to metastasize. Although uncommon in MIFS patients, there have been reports of cases of metastatic spread to lymph nodes, lungs, the neck, and the base of the skull 8, 9.

Here, we present a case of recurrent MIFS in the lower left extremity, with a biopsy‐proven recurrence at the proximal surgical margin, 2 years after his initial definitive surgical treatment. The patient's primary complaint at the time of recurrence was a changing skin lesion near his prior surgical incision site with associated tenderness.

## Case Report

A 74‐year‐old male patient with a history of a previously diagnosed myxoinflammatory fibroblastic sarcoma of the left lower extremity treated with definitive surgery presented <2 years later for the development a new tender area of nodularity distal to his previous surgical incision site. The patient's past medical history was also significant for early stage nonsmall cell cancer approximately 20 years prior, which was treated surgically, as well as localized prostate cancer treated with radical prostatectomy.

The patient had initially presented with a mass on the right anterior‐medial shin, which at the time measured approximately 4.5 cm. He subsequently underwent an excisional biopsy which revealed low‐grade myxoinflammatory fibroblastic sarcoma. One month later, the patient underwent a wide local excision and negative margins were achieved. The patient then underwent skin graft placement and close observation with no adjuvant therapy.

**Figure 1:**
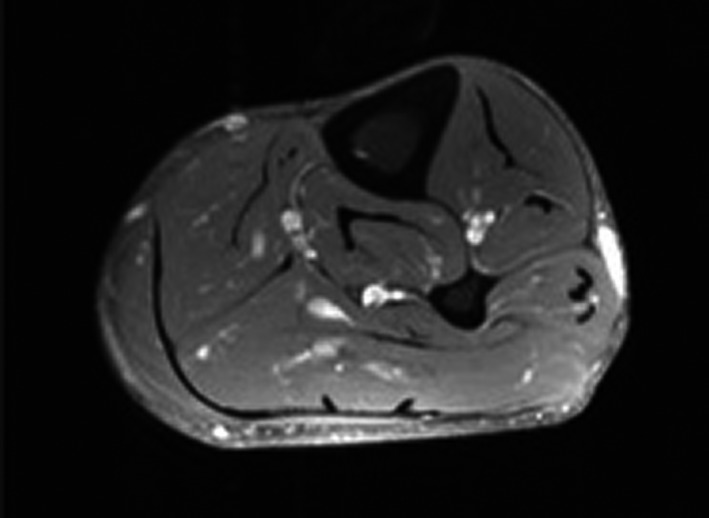
Axial T1‐weighted free spin magnetic resonance image of the left lower leg showing an avidly enhancing, ovoid‐shaped subcutaneous nodule. The nodule has a thin tail of enhancement extending posteriorly to the level of the surgical scar. The mass intimately abuts the superficial fascia of the peroneal musculature, without extension deep to the fascia.

Fourteen months later, the patient noted the development of a tender nodule on the anterior/inferior edge of his prior surgical scar. The patient underwent wide local excision with pathological evaluation showing recurrent myxoinflammatory fibroblastic sarcoma measuring 2.5 cm in size, with extension to the deep margin and a close 1 mm anterior margin. A metastatic workup including CT scans of the chest, abdomen, and pelvis revealed no evidence for metastatic progression of the tumor. An MRI scan 1 month following surgery revealed an “enhancing subcutaneous nodule on the lateral lower leg at the proximal surgical margin consistent with residual recurrent tumor” (Fig. 1). The tumor showed contact with the underlying superficial fascia of the lateral compartment although no discrete invasion, swelling, or dysfunction was present.

**Figure 2:**
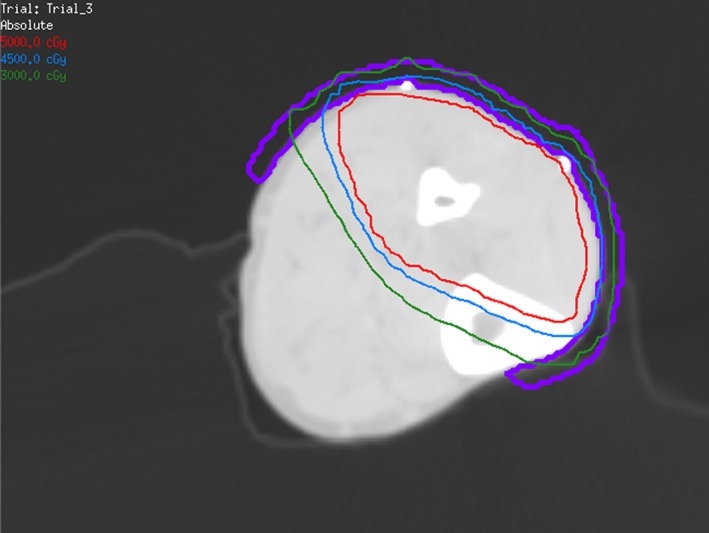
Axial computed tomography image showing radiotherapy isodose curves and bolus in the left lower leg.

**Figure 3:**
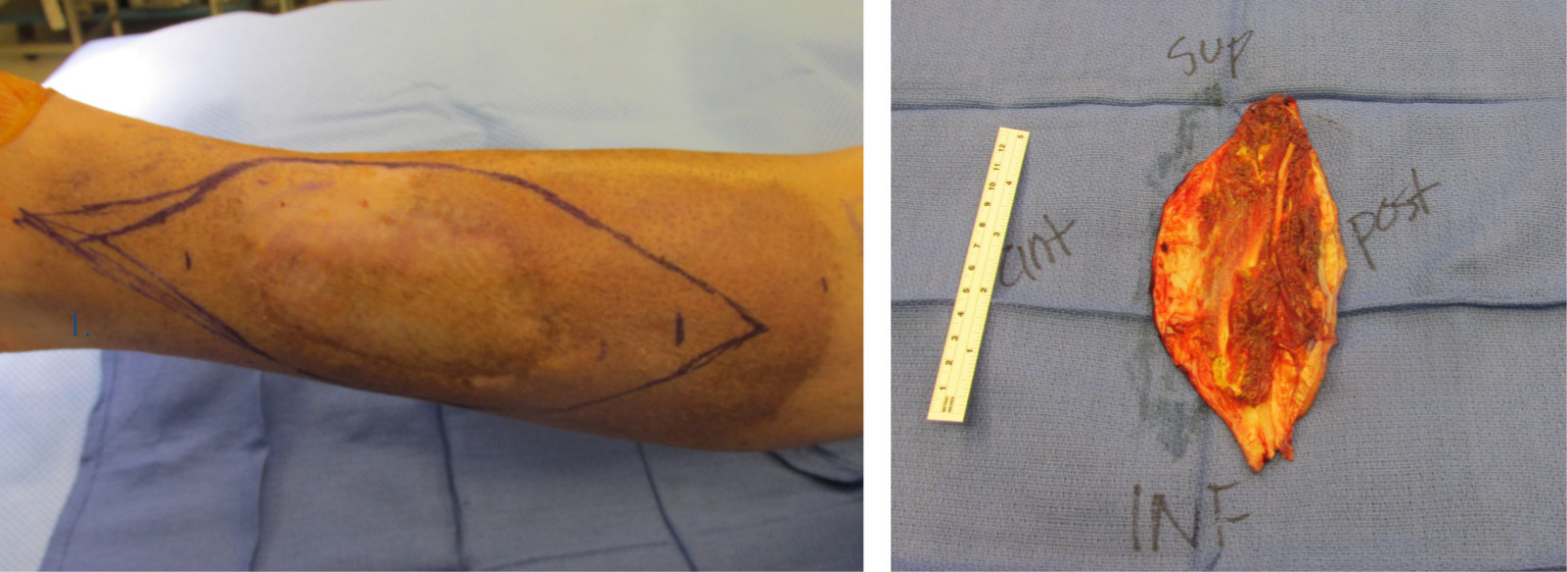
Preoperative photograph showing a central area of residual sarcoma, surrounding hyperpigmentation following external beam radiation therapy, and demarcation of planned surgical incision for adequate tumor margin. Postoperative photograph showing the orientation and posterior depth of invasion of the surgical specimen following wide local excision.

**Figure 4:**
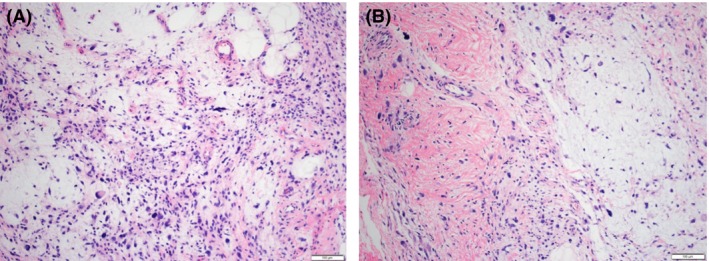
(A) High‐grade tumor with bizarre multinucleated giant tumor cells and cytologic atypia (10X). (B) High‐grade tumor with myxoid background, markedly atypical cells and scattered inflammatory cells (10X).

After multidisciplinary tumor board discussion, the decision was made to treat the patient with preoperative radiation therapy, followed by oncologic orthopedic surgery due to the recurrent nature of the patient's disease. The patient was prescribed a course of radiation therapy to a dose of 5000 cGy in 25 fractions with 6‐MV photons delivered with an intensity‐modulated radiation therapy technique (IMRT) (Fig. 2). This was in an effort to spare bone and reduce the risk of lymphedema as the patient had previously undergone multiple surgical procedures. The patient underwent a wide surgical resection and skin grafting procedure approximately 3 weeks after the completion of radiation therapy. The surgical resection encompassed a 2–3 cm margin around the patient's residual disease and included a portion of the anterior compartment, lateral compartment, and small portion of the gastrocnemius muscle posteriorly (Fig. 3). Microscopically, the tumor is composed of areas of solid sheets of atypical cells with marked nuclear pleomorphism. There are also areas with myxoid stroma. Scattered bizarre multinucleated giant cells are present. Rare mitotic figures are seen. There are no areas of necrosis (Fig. 4). The tumor had focal fascial invasion without underlying muscle involvement, and clear negative margins were achieved. Following wide surgical excision, the patient underwent a complex closure requiring split thickness skin grafting. At 3‐month follow‐up, the patient continues to recover from surgery, and postoperative imaging shows no evidence of metastatic disease on CT of the chest. MRI of the lower extremity shows interval postsurgical change with no evidence of nodular mass like signal abnormality or enhancement. Clinically, the patient remains neurologically intact in the lower extremity, with little‐to‐no evidence of lymphedema following surgery. He did experience some loss of his skin graft at the distal aspect requiring vacuum‐assisted closure and will soon undergo additional skin grafting to correct this defect.

## Discussion

Occurrences of myxoinflammatory fibroblastic sarcoma have been detailed in literature mostly through individual case reports and institutional case series. An institutional case series of five patients along with review of the literature in 2013 identified 138 cases following a MEDLINE literature review of cases from 1997 to 2012. This analytical review from the Rizzoli Orthopedic Institute highlighted various characteristics of MIFS. They identified pertinent clinic‐pathological data for these patients when present; however, many of the individual patient details were not available. Their data showed that the median age of patients was 45 years (34–56 years), with slightly more cases reported in males 55% (76/138 cases) versus 45% (62/138 cases) in females. The median tumor size reported was 3 cm (2–5 cm) 5. Of all cases analyzed, common sites of occurrence included the hand (24%), upper extremity digits (23%), and foot (20%). A minority of the patient with available data 17% (14/82 patients) reported feeling pain at diagnosis. The utilization of radiation therapy with either external beam radiation therapy (EBRT) alone or in combination with brachytherapy/intraoperative radiotherapy was limited. Neoadjuvant EBRT was utilized in 15 cases, while adjuvant EBRT usage was noted in only 12 cases. With a median follow‐up of 26 months, they noted a recurrence rate of 22% (26/118 patients), with a median time to recurrence was 15 months. Accounting for all recurrences, there were notably four cases (3%) of lymph node metastases or distant lung metastases 5. A retrospective case series from Massachusetts General Hospital reported on the outcome of 17 patients with MIFS and also indicated that MIFS more commonly affects middle‐aged adults with a higher prevalence in men 10.

Additionally, although MIFS has traditionally been overemphasized as being present in the distal extremities, the originally descriptive “acral” term for the tumor appears to be highly negligent of other common sites (hands and fingers) 1, 5. This correction is particularly important in light of the current case being studied, in which the recurrent tumor appeared on the left lower extremity. Similar to the current case being studied, the tumor appears as a swollen nodule in the subcutaneous tissue, and associated patient‐reported pain is unusual 5, 8, 11, 12.

Immunohistochemical studies showed that when testing for vimentin was performed, it was strongly positive in all lesions. Tumors often demonstrated focal positivity for CD68 (84%) and CD34 (57 %). Positivity for smooth muscle actin (SMA), S‐100 protein, activin receptor‐like kinase 1 (ALK‐1), and keratin was less common (6–16 %). No cases showed positive staining for CD30, leukocyte common antigen (LCA), desmin, epithelial membrane antigen (EMA), HMB45, or melan‐A. Although less frequently utilized, immunoreactivity rates for *α*1‐ACT, *α*1‐AT, CD95 and CD117 may also be high for myxoinflammatory fibroblastic sarcoma 5.

Literature suggests that the most common methods of treatment are surgery, including both local excision and amputation, with the latter being cited as the “only predictive factor of clear margins” 5. Such extreme measures may be the result of inadequate excision of the tumor, as clinical diagnoses oftentimes incorrectly indicate a benign disease 5. However, surgery is maintained as the primary method of treatment because of past successful curative history of surgical excision 5.

Local recurrence is the primary problem in cases of MIFS posttreatment. One recent database review population of 138 cases showed a local recurrence rate of 22% alongside a five year relapse‐free survive (RFS) probability of 67% 5. Margins after surgery showed clear margins in 81% (26/32) of patients and marginal/intralesional in 6% (2/32) of patients 5. There was no report of margins in 13% of patients. First recurrences were local in 25 patients, with local and lymph node metastasis present in only one patient 5.

Another survey of all published cases of this disease reported a crude local recurrence rate of 31.3%; however, the majority of these patients were treated with surgery alone 10. The scarcity of published reports on such cases increases the variability of this statistic. Additionally, other measures of treatment, particularly using radiation, have only been studied in a limited number of cases. In the case series by the Massachusetts General Hospital, only one case of seventeen patients experienced local recurrence after the use of perioperative radiotherapy. The case of recurrence was in a patient with previously recurrent disease following definitive surgical treatment 10. Similar to their described case of recurrent disease, our patient will be at a higher risk for recurrence despite XRT.

In our case, the decision to proceed with radiotherapy was based primarily on the presence of the patient's two previous recurrences following surgical excision without adjuvant therapy. The morbidity related to radiotherapy treatment in the distal extremity is low and should be well tolerated with the utilization of an IMRT technique. Our patient's multiple recurrent MIFS has shown he is at risk for additional recurrence, which as suggested by the MGH data can be decreased with the usage of preoperative radiation therapy.

The rarity of this disease and the incomplete nature of case reports and studies regarding the development of MIFS tumors complicate the development of definitive conclusions regarding the best methods of treatment for this disease as well as the potential for local recurrences and metastases. There are suggestions that radiotherapy may have a role in improving local control, especially in cases involving positive surgical margins, and potentially in recurrent disease 10. The variations in treatment plans across hospitals and institutions that have dealt with this illness further confound the implications of the statistics and findings analyzed and presented in this paper.

## Conclusion

Case reports detailing the existence of myxoinflammatory fibroblastic sarcoma currently remain limited. Those cases have been predominately treated with a definitive surgical approach; however, the usage of radiotherapy is also increasing in the setting of difficult surgical excisions or the high likelihood of local recurrence. Case reports analyzing occurrences of MIFS detail a range of target areas throughout the body, but the disease does notably show a predilection for the hands and feet. The limited number of reported cases and a lack of available data for many of the treatment plans and criteria for diagnoses of MIFS make it difficult to draw definitive conclusions regarding the best methods for detection and treatment of the disease. The use of FISH and immunohistological staining can help in making proper diagnoses of such abnormal cells in rarely seen tumors. Nonetheless, a greater amount of data on as many aspects of this illness in a multitude of patients is necessary to further proper diagnosis and treatment of MIFS.

## Authorship

NJ: involved in conception or design of the work, data collection, drafting the article, critical revision of the article, and final approval of the version to be published. DHM: involved in conception or design of the work, data collection, drafting the article, critical revision of the article, and final approval of the version to be published. WS: involved in conception or design of the work, data collection, drafting the article, critical revision of the article, and final approval of the version to be published. SA: involved in drafting the article, critical revision of the article, and final approval of the version to be published. CES: involved in drafting the article, critical revision of the article, and final approval of the version to be published. CC: involved drafting the article, critical revision of the article, and final approval of the version to be published. BCM: involved in conception or design of the work, data collection, drafting the article, critical revision of the article, and final approval of the version to be published. RCM: involved in conception or design of the work, data collection, drafting the article, critical revision of the article, and final approval of the version to be published.

## Conflict of Interest

None declared.
